# Induction of tetraploids in Paper Mulberry (*Broussonetia papyrifera* (L.) L’Hér. ex Vent.) by colchicine

**DOI:** 10.1186/s12870-023-04487-2

**Published:** 2023-11-17

**Authors:** Jiana Lin, Bingnan Zhang, Jintuo Zou, Zhen Luo, Hao Yang, Peng Zhou, Xiaoyang Chen, Wei Zhou

**Affiliations:** 1 State Key Laboratory for Conservation and Utilization of Subtropical Agro-Bioresources (South China Agricultural University), Guangzhou, 510642 China; 2 Guangdong Key Laboratory for Innovative Development and Utilization of Forest Plant Germplasm, Guangzhou, 510642 China; 3 Guangdong Province Research Center of Woody Forage Engineering Technology, Guangzhou, 510642 China; 4 College of Forestry and Landscape Architecture, South China Agricultural University, Guangzhou, 510642 China; 5 Guangdong Engineering Technology Research Center of Agricultural and Forestry Biomass, South China Agricultural University, Guangzhou, 510642 China; 6 Guangdong Eco-Engineering Polytechnic, Guangzhou, 510642 China

**Keywords:** *Broussonetia papyrifera*, Polyploid, Colchicine, Photosynthetic features

## Abstract

**Background:**

*Broussonetia papyrifera* (L.) L’Hér. ex Vent. has the characteristics of strong stress resistance, high crude protein content, and pruning tolerance. It is an ecological, economic, and medicinal plant. Polyploid plants usually perform better than their corresponding diploid plants in terms of nutrients, active substances, and stress resistance.

**Results:**

In this study, the leaves, calli, and seeds of diploid *B. papyrifera* were used for tetraploid induction by colchicine. The induction effect of colchicine on *B. papyrifera* was summarized through the early morphology, chromosome count and flow cytometry. It was concluded that the best induction effect (18.6%) was obtained when the leaves of *B. papyrifera* were treated in liquid MS (Murashige and Skoog) medium containing 450 mg·L^-1^ colchicine for 3 d. The comparative analysis of the growth characteristics of diploid and tetraploid *B. papyrifera* showed that tetraploid *B. papyrifera* has larger ground diameter, larger stomata, thicker palisade tissue and thicker sponge tissue than diploid *B. papyrifera*. In addition, the measurement of photosynthetic features also showed that tetraploids had higher chlorophyll content and higher photosynthetic rates.

**Conclusion:**

This study showed that tetraploid *B. papyrifera* could be obtained by treating leaves, callus and seeds with liquid and solid colchicine, but the induction efficiency was different. Moreover, there were differences in stomata, leaf cell structure and photosynthetic features between tetraploid *B. papyrifera* and its corresponding diploid. The induced tetraploid *B. papyrifera* can provide a technical basis and breeding material for the creation of *B. papyrifera* germplasm resources in the future.

**Supplementary Information:**

The online version contains supplementary material available at 10.1186/s12870-023-04487-2.

## Background

Polyploidy refers to having more than two sets of chromosomes in each cell, and most polyploid chromosomes are even [1]. Polyploidy plays a key role in promoting phenotypic diversity and evolution [2, 3]. Fawcett et al. [4] found that many plant lineages had independent genome-wide replication events in the Cretaceous-Tertiary (KT) [5]. Nonetheless, previous estimates on the frequency of polyploid species formation have shown that the formation and establishment of new polyploid species in nature are rare [6]. The genetic diversity of polyploids increased relative to their diploid ancestors. In addition, this genetic diversity may show novelty at the biochemical, physiological, morphological and ecological levels, so polyploids are better than diploid parents at least in the short term [7]. The increase of polyploid genetic variation may lead to increased tolerance to a wider range of ecological and environmental conditions. For example, the total seed yield of tetraploid *Themeda triandra* Forsk is more than four times higher than that of diploids under drought and high-temperature stress [8]. Nowadays, global climate anomalies are frequent, and it is also a good strategy to improve the adaptability of plants by inducing polyploidy [9, 10]. Moreover, compared with diploid plants, polyploid plants have larger vegetative storage organs [11], higher contents of active substances [12, 13] and stronger disease [14]. For example, Xi et al. [15] found that polyploid *Populus tomentosa* grew rapidly in North China, and its 8-year volume was 2–3 times that of diploid control. Hu et al. [16] found that triploid and tetraploid carambola produced thicker and larger leaves, larger pollen grains and flowers, and larger fruits than diploid carambola; by comparing the differences between diploid and polyploid Rhododendron, Mo et al. [17] concluded that the leaves of polyploid Rhododendron were larger and thicker; Hias et al. [18] based on visual symptom assessment and real-time PCR to quantify the DNA of apple scab in apple leaves, and observed that tetraploid apples with a single gene resistant genotype had increased resistance compared to their diploids.

Ploidy breeding is the breeding process of obtaining offspring with changed chromosome multiples through artificial mutation technology. It has been widely used in breeding new varieties to improve their value [19]. For example, it can improve the content of active ingredients in some medicinal plants [20, 21] and the fruit quality of some fruit crops [22]. Polyploidy can be induced by sexual hybridization or somatic chromosome doubling. Sexual hybridization is based on the principle that male or female parents can produce unreduced gametes (2n). The main obstacle to the application of sexual polyploidy is the low frequency of unreduced gametes [23]. In contrast, somatic chromosome doubling is more widely used in plant polyploid breeding, which can be induced by extremely high or low-temperature upheaval, ionizing radiation, chemical reagents and so on. Among them, colchicine induction polyploidy is one of the common induction methods. Colchicine is a tubulin inhibitor and microtubule interfering agent. The principle of colchicine-induced polyploidy is to combine with heterodimers in mitotic cells, hinder the formation of spindle filaments, destroy spindle function, and inhibit the movement of chromosomes to the two poles of cells to form chromosome-doubled cells [24, 25]. Many plants have successfully induced polyploidy with colchicine. For example, with colchicine concentration of 150 mg·L^−1^ for 7 d, induced *Zingiber Officinale* Roscoe cv. ‘Fengtou’ ginger with chromosome doubling rate reached 18% [26]; 0.1%(w/v) colchicine for 48 h effectively induced polyploidy in *Hyoscyamus reticulatus* L. [27]; the seeds of *Acacia mearnsii* were treated with 0.01% colchicine for 6 h and successfully induced tetraploid [28].

*B. papyrifera* is a deciduous tree belonging to the mulberry family, which is widely distributed in Asia and the Pacific [29]. Its phloem fiber is long and has been a good material for papermaking since ancient times [30]. *B. papyrifera* contains flavonoids, terpenoids, alkaloids and other bioactive substances, which have antioxidant, antibacterial, anti-inflammatory and other effects [31–34]. In recent years, more and more scholars have found that *B. papyrifera* is a woody plant with high protein, high fat and low crude fiber, which has the potential to become an excellent feed raw material. For example, the production performance and meat quality of Hu sheep were improved after feeding with the feed added with *B. papyrifera* fermentation [35]. Adding *B. papyrifera* silage can enhance the immune and antioxidant functions of Holstein cows [36]. *B. papyrifera* grows rapidly and has strong stress resistance [37] and may adapt to various adverse conditions of heavy metal-polluted soil and karst soils [38]. Therefore, it is a good tree species for ecological restoration and the greening of urban settings [39].

In this study, the leaves, callus and seeds of diploid *B. papyrifera* were used as explant materials to induce polyploidy under different concentrations of colchicine and treatment durations, and the artificially induced tetraploid *B. papyrifera* was obtained for the first time. Meanwhile, by comparing the morphological characteristics and physiology of diploid and tetraploid *B. papyrifera*, we can more comprehensively understand the differences between *B. papyrifera* of different ploidies. The purpose of this study was to obtain polyploid *B. papyrifera* to improve its biomass, and content of protein and improve its feeding value and ecological restoration ability.

## Materials and methods

### Plant material and growth conditions

The diploid seeds of *B. papyrifera* were from South China Agricultural University (23° N, 113° E). Mature and healthy *B. papyrifera* seeds were selected, eroded in concentrated sulfuric acid for 9 min to soften the seed coat, then treated with 70% ethanol for 50 s and 2% sodium hypochlorite for 15 min, and rinsed with sterile water for 3–4 times. After that, put the seeds into a sterile seed culture medium and cultivated into plantlets in a tissue culture room. The leaf material comes from the leaves of sterile *B. papyrifera* seedlings, and the callus is induced from the leaves of seedlings in the MS (Murashige and Skoog) + 2.0 mg·L^−1^ 6BA (6-benzyladenine) + 0.05 mg·L^−1^ IBA (indole-3-butyric acid).

The solid colchicine medium was colchicine + MS + 2.0 mg·L^−1^ 6BA + 0.05 mg·L^−1^ IBA + 3% (w/v) sucrose + 0.6% (w/v) agar for adventitious shoot induction, and colchicine + 1/2 MS + 3% (w/v) sucrose + 0.4% (w/v) agar for seed culture. A liquid medium was used to remove agar based on a solid medium. All adventitious shoots induced root formation in rooting medium 1/2 MS (Half-strength Murashige and Skoog) + 0.05 mg·L^−1^ NAA (α-naphthalene acetic acid) + 3% (w/v) sucrose + 0.6% (w/v) agar. The pH of all media was adjusted to 5.8 – 6.2 with HCl or NaOH solution. Colchicine was purchased from Sigma-Aldrich (St. Louis, MO, USA), the plant growth regulators were purchased from Phyto Technology Laboratories (Lenexa, Kansas, USA), and agar, sucrose, concentrated sulfuric acid and hydrochloric acid were obtained from the Guangzhou reagent factory (Guangzhou, Guangdong). The materials were cultured in the tissue culture room with a 26 ± 2℃, 1500 lx light intensity and 12 h light/dark photoperiod.

### Polyploidy induction and identification

#### Induction treatment of solid colchicine medium

Under aseptic conditions, the leaves of sterile *B. papyrifera* plantlets close to the stem tip were cut into 1.0 × 1.0 cm^2^ pieces, and the calli were cut into 1 × 1 × 1 cm^3^ pieces. The leaf pieces were precultured on MS medium for 3 d and then transferred to adventitious shoot induction medium supplemented with colchicine (0, 250, 350, 450, 550 mg·L^−1^) for 1, 2, 3, and 4 w in the dark. The calli were cultured in the dark for 1, 2, 3, and 4 w in the adventitious shoot induction medium supplemented with 0, 150, 250, 350 and 450 mg·L^−1^ colchicine. After colchicine treatment, the explants were transferred to an adventitious shoot induction medium without colchicine for normal culture. After 45 d, the number of induction adventitious shoot was counted and then the adventitious shoots were cut off and transferred to the rooting medium. When the plantlet height reached approximately 10 cm, its ploidy was identified, and the optimum time of tetraploid induction was recorded.

#### Induction treatment of liquid colchicine medium

The cut leaves were placed into the liquid medium for adventitious shoot induction with colchicine concentrations of 0, 250, 350, 450 and 550 mg·L^−1^, while the calli were induced in liquid medium with 0, 150, 250, 350 and 450 mg·L^−1^ colchicine. Treatments were bred for 1, 2, 3 and 4 d with continuous shaking at 80 rpm in the dark and then washed with sterile water. Subsequently, the treated explants were transferred to a solid adventitious shoot induction medium without colchicine. The number of induction adventitious shoot was counted after 45d. When the adventitious shoot grew to approximately 2 cm, they were transferred to the rooting medium.

#### Soak the seeds with colchicine

Put the sterilized seeds into the seed culture medium, when the seeds grew 2 mm radicle, they have soaked in colchicine solutions with concentrations of 0, 100, 200, 300 and 400 mg·L^−1^ for 1, 2 and 4 d. Thereafter, the seeds were washed with sterile water 3–4 times and then inoculated into the seed culture medium.

#### Chromosome counting

According to the method of Chen et al. [40], the chromosome number of the root tip was counted, and the chromosome number of tetraploid plants and control diploid plants was determined. At about 10:00 am, the root tips were cut from the regenerated plantlets and collected for chromosome counting. The samples were pretreated with ice water (4 °C) for 10 h and then fixed in fresh Carnoy’s solution (glacial acetic acid: 95% ethanol, 1:3) for 24 h. After the fixed root tips were rinsed with water for 15 min, the root tips were treated with 1 N HCl at a constant temperature of 60 °C for 8 min and then rinsed with water for 20 min. Root tip meristems about 1 mm long were stained with Modified Carbol-Fuchsin Solution (Solarbio, Beijing, China) for 20 min and then squashed on a microscope slide, and the chromosome number was observed with a microscope (DS-Ri2, Nikon, Tokyo, Japan).

#### Flow cytometry analysis

The DNA content of leaf cells in colchicine-treated and control diploid *B. papyrifera* (the control diploid *B. papyrifera* was used as the internal standard.) was measured by flow cytometry (Sysmex CyFlow® Ploidy Analyzer, Görlitz, Germany). The kit was a CyStain® UV Precise P (JIYUAN BIO-TECH, China). Fresh tender leaves were harvested, cut into 0.5 × 0.5 cm^2^, and vertically chopped with a blade in 400 μL extraction buffer for 30–60 s. After filtration through a 30 μm filter membrane, the samples were stained with 1600 μL 4',6-diamidino-2-phenylindole and dihydrochloride (DAPI) for 2 min and then tested on the machine.

### Comparison of different ploidies of *B. papyrifera*

#### Morphological analysis

The regenerated plantlets of diploid and tetraploid *B. papyrifera* were transplanted into the substrate (perlite: peat soil = 1:1) and grown in the greenhouse (the temperature was controlled at 26 ± 2℃, and the light source was natural light) for two months. The plant height, ground diameter, leaf length, leaf width, petiole length and internode distance of tetraploid and diploid plantlets were measured.

#### Observation of stomatal and leaf cell structure

Tweezers were used to tear out the transparent epidermis of *B. papyrifera* leaves of different ploidy levels with the same plantlet stage and quickly placed into the water droplets on the prepared glass slide to make a temporary water sealing sheet. After production, 10 visual fields were observed and photographed under a microscope (DS-Ri2, Nikon, Tokyo, Japan), and the length of the stomata was measured.

The production of paraffin sections of leaves involves cutting the leaves into small pieces, fixing them with FAA (Formaldehyde Alcohol Acetic Acid) Fixative immediately for 24 h, embedding the materials with paraffin and slicing them. The sections were observed with a microscope (DS-Ri2, Nikon, Tokyo, Japan), and the thicknesses of the upper epidermis, lower epidermis, palisade tissue and sponge tissue were measured and photographed.

#### Photosynthetic features

From 9:30 am to 12:30 am on a sunny day, the second leaf of diploid and tetraploid plantlets was selected from top to bottom to measure the data of photosynthetic features. The chlorophyll content was measured by a portable chlorophyll content meter (SPAD 502 Plus, Osaka, Japan), and the net photosynthetic rate (Pn, μmol·m^−2^·s^−1^), stomatal conductance (Gs, mol·H_2_Om^−2^·s^−1^), intercellular CO_2_ concentration (Ci, μl·L^−1^) and transpiration rate of leaves (Tr, mmol·m^−2^·s^−1^) were measured by Portable photosynthetic apparatus LI-6400XT (Li-Cor BioScience, Lincoln, NE, USA), and the light intensity was set to 1000 μmol·s^−1^.

### Statistical analyses

Each colchicine induction treatment was repeated 3 times, and each repetition contained 15 experimental materials. The number of explants inducing buds was counted and the induction rate of the adventitious shoot was calculated. All the adventitious roots were analyzed by flow cytometry after chromosome observation and counting. Then according to the results, the chimera induction rate and tetraploid induction rate were counted. Data were analyzed by using SPSS version 25.0 (SPSS, Inc., Chicago, IL, USA) and Excel (Microsoft Corp., Redmond, WA, USA).

$$\text{Adventitious shoot induction rate}\ (\%)=\frac{\text{Number of budding explants}}{\text{Number of explants}}\times100$$

$$\text{Seed survival rate}\ (\%)=\frac{\text{Number of germinated seeds}}{\text{Number of seeds}}\times100$$

$$\text{Tetraploid induction rate}\ (\%)=\frac{\text{Number of tetraploid plantlets}}{\text{Number of adventitious shoots}}\times100$$

$$\text{Mixed ploidy induction rate}\ (\%)=\frac{\text{Number of chimeric plantlets}}{\text{Number of adventitious shoots}}\times100$$

$$\text{CTR}\ (\text{cell tension ratio})\ (\%)=\frac{\text{Thickness of palisade tissue}}{\text{Thickness of leaf}}\times100$$

## Results

### Tetraploid induction and verification

#### Induction efficiency of solid colchicine medium

Table 1 and Table S1 showed that there were differences in adventitious shoot induction of *B. papyrifera* leaves under the conditions of different colchicine concentrations and treatment times. When the concentration of colchicine was constant, the induction rate of adventitious shoot decreased gradually with increasing treatment time. When the concentration of colchicine was 350 mg·L^−1^ and the treatment time was 3 w, the induction rate of tetraploids was the highest, which was 13.6%. Table 2 (Table S2) indicates that although the adventitious shoot induction rate of the callus was high under the treatment of a solid colchicine medium, tetraploids were not induced.

**Table 1:** Effect of solid colchicine medium on leaf induced polyploidy

Colchicine Concentration (mg·L^−1^)	Duration (w)	Adventitious shoot induction rate (%)	Mixed ploidy induction rate (%)	Tetraploid induction rate (%)
0	0	95.6 ± 2.2a	0b	0b
250	1	93.3 ± 4.4ab	0b	0b
250	2	80.0 ± 3.8bcde	3.4 ± 1.7ab	0b
250	3	73.3 ± 10.2cdefg	4.2 ± 2.1ab	2.0 ± 2.0b
250	4	68.9 ± 2.2efgh	8.3 ± 8.3ab	6.7 ± 6.7ab
350	1	86.7 ± 6.7abc	3.1 ± 1.6ab	2.8 ± 1.4b
350	2	77.8 ± 2.2cdef	9.10 ± 2.2ab	6.9 ± 0.4ab
350	3	64.4 ± 4.4fgh	13.9 ± 3.2a	13.6 ± 2.4a
350	4	48.9 ± 2.2ij	11.1 ± 11.1ab	6.7 ± 6.7ab
450	1	84.4 ± 5.9abcd	7.7 ± 0.8ab	7.2 ± 3.7ab
450	2	75.6 ± 2.2cdefg	3.7 ± 3.7b	3.3 ± 3.3ab
450	3	62.2 ± 2.2ghi	0b	0b
450	4	44.4 ± 4.4jk	0b	0b
550	1	71.1 ± 2.2defg	5.6 ± 5.6ab	0b
550	2	55.6 ± 2.2hij	0b	0b
550	3	42.2 ± 4.4jk	0b	0b
550	4	31.1 ± 5.9k	0b	0b

Each value represents the mean ± SE of three replicates, and means followed by the same letter in the same column are not significantly different from each other at *P* ≤ 0.05 level, according to Duncan’s multiple range test

**Table 2:** Effect of solid colchicine medium on callus induced polyploidy

Colchicine Concentration (mg·L^−1^)	Duration (w)	Adventitious shoot induction rate (%)	Mixed ploidy induction rate (%)	Tetraploid induction rate (%)
0	0	95.6 ± 2.2a	0b	0
150	1	91.1 ± 5.9ab	0b	0
150	2	86.7 ± 3.8abc	0b	0
150	3	86.7 ± 3.8abc	0b	0
150	4	82.2 ± 4.4abcd	0b	0
250	1	88.9 ± 11.1ab	0b	0
250	2	86.7 ± 10.2abc	0b	0
250	3	80.0 ± 7.7abcd	4.3 ± 2.1ab	0
250	4	71.1 ± 8.0abcd	10.8 ± 5.5a	0
350	1	82.2 ± 9.7abcd	0b	0
350	2	75.6 ± 5.9abcd	6.8 ± 0.9ab	0
350	3	68.9 ± 2.2bcd	9.26 ± 4.9ab	0
350	4	60.0 ± 10.2d	4.8 ± 4.8ab	0
450	1	73.3 ± 6.7abcd	4.6 ± 2.4ab	0
450	2	68.9 ± 2.2bcd	4.4 ± 4.4ab	0
450	3	62.2 ± 14.6cd	9.7 ± 5.8ab	0
450	4	57.8 ± 8.9d	0b	0

Each value represents the mean ± SE of three replicates, and means followed by the same letter in the same column are not significantly different from each other at *P* ≤ 0.05 level, according to Duncan’s multiple range test

#### Induction efficiency of liquid colchicine medium

Browning occurred in some materials treated with liquid colchicine during regeneration, and the induction rate of the adventitious shoot was low, but the ploidy induction rate was high. The results (Table 3, Table S3) indicated that when the leaves were treated with 450 mg·L^−1^ liquid colchicine for 3 d, the tetraploid induction rate was the largest, which was 18.7%. Although the adventitious shoot induction rate was higher when the colchicine concentration was 250 mg·L^−1^ and the treatment time was less than 3 d, tetraploidy was not induced. It can be seen from Table 4 (Table S4) that the induction rate of tetraploids obtained by treating the *B. papyrifera* callus with 350 mg·L^−1^ liquid colchicine for 2 d was the highest at 10.0%.

**Table 3:** Effect of liquid colchicine medium on leaf induced polyploidy

Colchicine Concentration (mg·L^−1^)	Duration (d)	Adventitious shoot induction rate (%)	Mixed ploidy induction rate (%)	Tetraploid induction rate (%)
0	0	95.6 ± 2.2a	0b	0b
250	1	93.3 ± 3.8a	0b	0b
250	2	86.7 ± 7.7ab	1.3 ± 1.3b	0b
250	3	84.4 ± 9.7ab	4.6 ± 2.4ab	2.6 ± 2.6b
250	4	77.8 ± 8.0ab	6.1 ± 3.0ab	3.3 ± 3.3b
350	1	91.1 ± 5.9a	3.7 ± 2.1b	1.2 ± 1.2b
350	2	84.4 ± 4.4ab	4.9 ± 2.9ab	3.2 ± 1.6b
350	3	75.6 ± 5.9abc	6.7 ± 3.4ab	3.7 ± 3.7b
350	4	68.9 ± 5.9bcd	8.3 ± 8.3ab	5.5 ± 5.6b
450	1	84.4 ± 8.0ab	8.0 ± 2.3ab	4.3 ± 2.2b
450	2	80.0 ± 3.8ab	13.1 ± 2.6ab	10.4 ± 6.5ab
450	3	57.8 ± 2.2cd	19.2 ± 4.8a	18.7 ± 3.2a
450	4	53.3 ± 3.8d	11.1 ± 11.1ab	8.3 ± 8.3ab
550	1	82.2 ± 8.0ab	7.0 ± 3.9ab	4.4 ± 4.4b
550	2	75.6 ± 4.4abc	6.4 ± 3.2ab	3.0 ± 3.0b
550	3	55.6 ± 8.0d	4.6 ± 4.8ab	0b
550	4	51.1 ± 5.9d	0b	0b

Each value represents the mean ± SE of three replicates, and means followed by the same letter in the same column are not significantly different from each other at *P* ≤ 0.05 level, according to Duncan’s multiple range test

**Table 4:** Effect of liquid colchicine medium on callus induced polyploidy

Colchicine Concentration (mg·L^−1^)	Duration (d)	Adventitious shoot induction rate (%)	Mixed ploidy induction rate (%)	Tetraploid induction rate (%)
0	0	95.6 ± 2.2a	0d	0c
150	1	91.1 ± 5.9ab	0d	0c
150	2	88.9 ± 5.9abc	4.7 ± 0.8bcd	0c
150	3	80.0 ± 7.7abcd	7.0 ± 1.9bcd	0c
150	4	73.3 ± 3.8abcde	8.3 ± 3.8bcd	3.0 ± 1.5bc
250	1	91.1 ± 4.4abc	3.3 ± 0.7cd	0c
250	2	84.4 ± 9.7abcd	5.7 ± 0.8bcd	2.3 ± 1.2bc
250	3	71.1 ± 13.5abcde	9.0 ± 3.0bcd	4.0 ± 2.0bc
250	4	62.2 ± 8.9cdef	11.7 ± 2.5bcd	7.0 ± 4.1ab
350	1	84.4 ± 12.4abcd	7.3 ± 1.2bcd	0c
350	2	68.9 ± 8.9abcde	15.3 ± 3.2bc	10.0 ± 0.6a
350	3	60.0 ± 10.2def	31.7 ± 1.6a	3.7 ± 3.7bc
350	4	51.1 ± 9.7ef	16.3 ± 8.5b	0c
450	1	64.4 ± 11.8bcdef	12.3 ± 2.4bc	5.0 ± 2.5abc
450	2	57.8 ± 8.0def	10.3 ± 5.4bcd	0c
450	3	48.9 ± 5.9ef	6.7 ± 6.7bcd	0c
450	4	40.0 ± 6.7f	0d	0c

Each value represents the mean ± SE of three replicates, and means followed by the same letter in the same column are not significantly different from each other at *P* ≤ 0.05 level, according to Duncan’s multiple range test

#### Soaking the seeds with colchicine

Although the survival rate of seeds treated with 100 mg·L^−1^ and 200 mg·L^−1^ liquid colchicine was relatively high, the chimerism induction rate and tetraploid induction rate were very low. The tetraploid induction rate was the highest (11.7%) in the 4 d and 400 mg·L^−1^ treatments, although in this treatment, the survival rate decreased by 22.2%, and the radicle was expanded, the root hair was thick, and the growth was slow (Table 5, Table S5).

**Table 5:** Polyploidy induced by soaking seeds in liquid colchicine

Colchicine Concentration (mg·L^−1^)	Duration (d)	Germination rate (%)	Mixed ploidy induction rate (%)	Tetraploid induction rate (%)
0	0	97.8 ± 2.2a	0b	0b
100	1	86.7 ± 3.9ab	0b	0b
100	2	77.6 ± 0.5b	0b	0b
100	4	68.9 ± 2.2bc	0b	0b
200	1	80.0 ± 0b	0b	0b
200	2	68.9 ± 4.4bc	3.3 ± 3.3ab	0b
200	4	57.8 ± 5.9cd	7.4 ± 3.7ab	0b
300	1	71.1 ± 11.8bc	3.7 ± 3.7ab	0b
300	2	57.8 ± 5.9cd	5.5 ± 2.9ab	5.0 ± 2.8ab
300	4	44.4 ± 5.9de	8.9 ± 4.5ab	8.0 ± 4.0ab
400	1	51.1 ± 8.0d	4.7 ± 4.7ab	0b
400	2	33.3 ± 3.9ef	9.6 ± 5.0ab	8.6 ± 4.3ab
400	4	22.2 ± 2.2f	14.9 ± 7.6a	11.7 ± 7.3a

Each value represents the mean ± SE of three replicates, and means followed by the same letter in the same column are not significantly different from each other at *P* ≤ 0.05 level, according to Duncan’s multiple range test

#### Ploidy identification

**Fig. 1:**
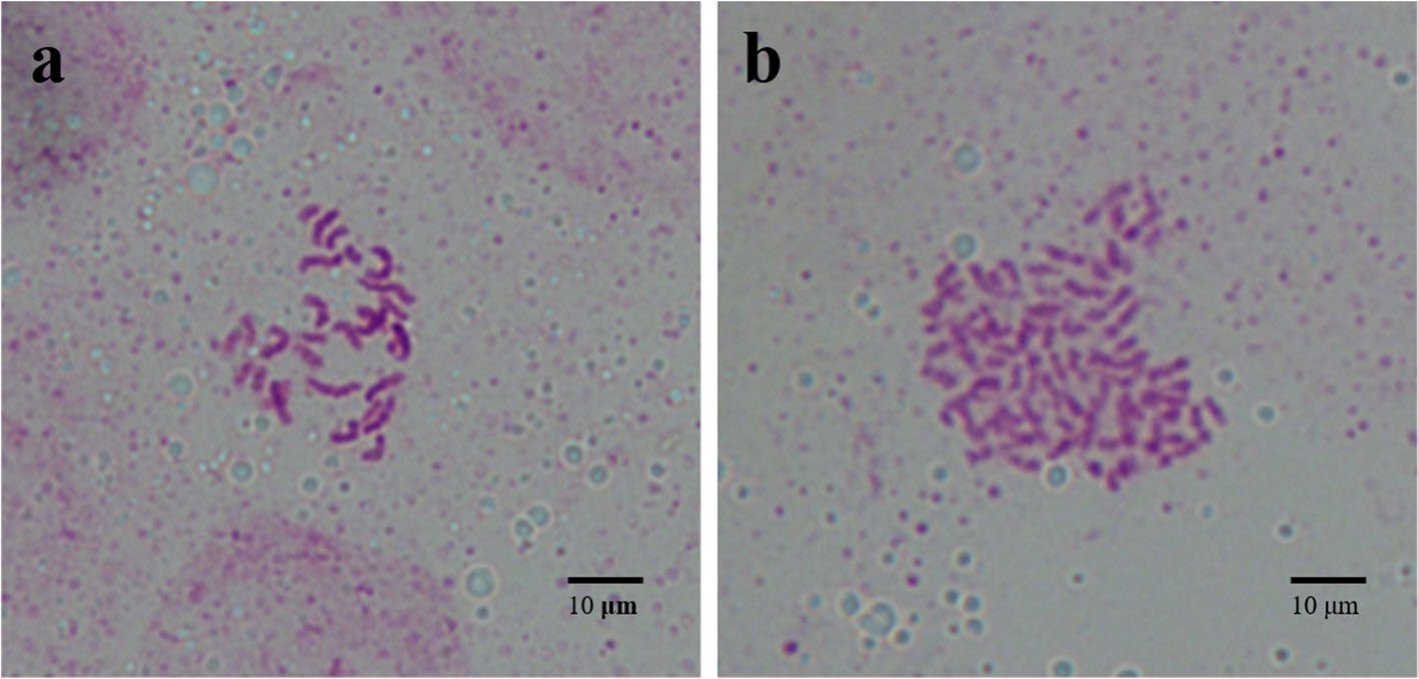
Chromosome count of diploid and tetraploid. **a** diploid; **b** tetraploid

Chromosome counting is an intuitive method to identify ploidy, which can directly observe the number of plant chromosomes. The results showed that the chromosome number of root tip cells of diploid control plants was 2*n* = 2*x* = 26 (Fig. 1a), while that of induced tetraploid plants was 2*n* = 4*x* = 52 (Fig. 1b).

**Fig. 2:**
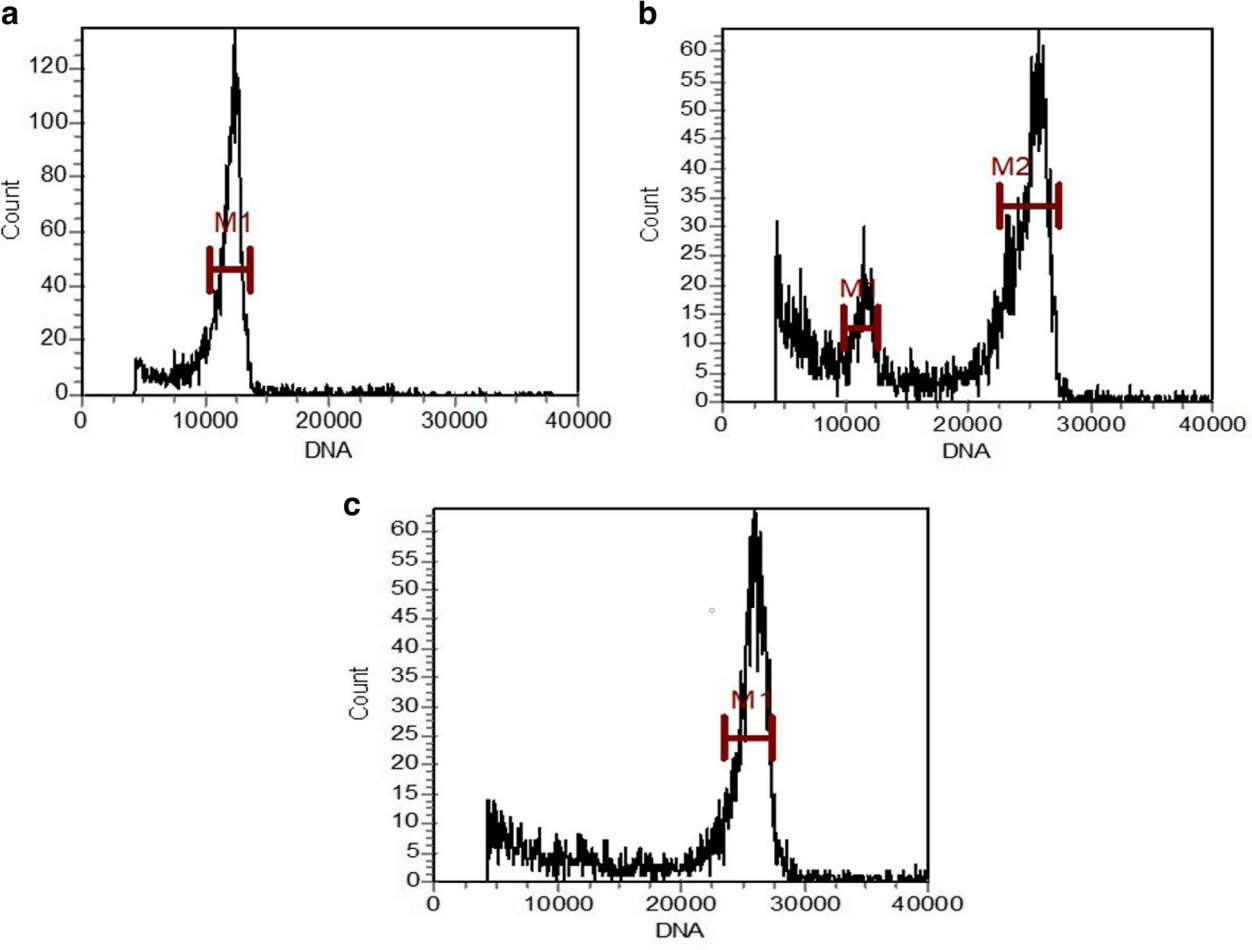
Flow cytometry results of diploid, mixed ploid and tetraploid. **a** diploid; **b** mixed ploid; **c** tetraploid, the diploid is the reference standard

The detection results of the relative content of leaf nuclei of diploid, mixed ploid and tetraploid plants by flow cytometry showed that the main peak of diploid appeared at about 13,000 (Fig. 2a), the mixed ploids had two peaks, 13,000 and 26,000 respectively (Fig. 2b). And the main peak of tetraploid appeared at about 26,000 (Fig. 2c). The results of tetraploid induction showed that the tetraploid trees were obtained.

### Comparison of diploid and tetraploid characteristics

#### Morphological

**Fig. 3:**
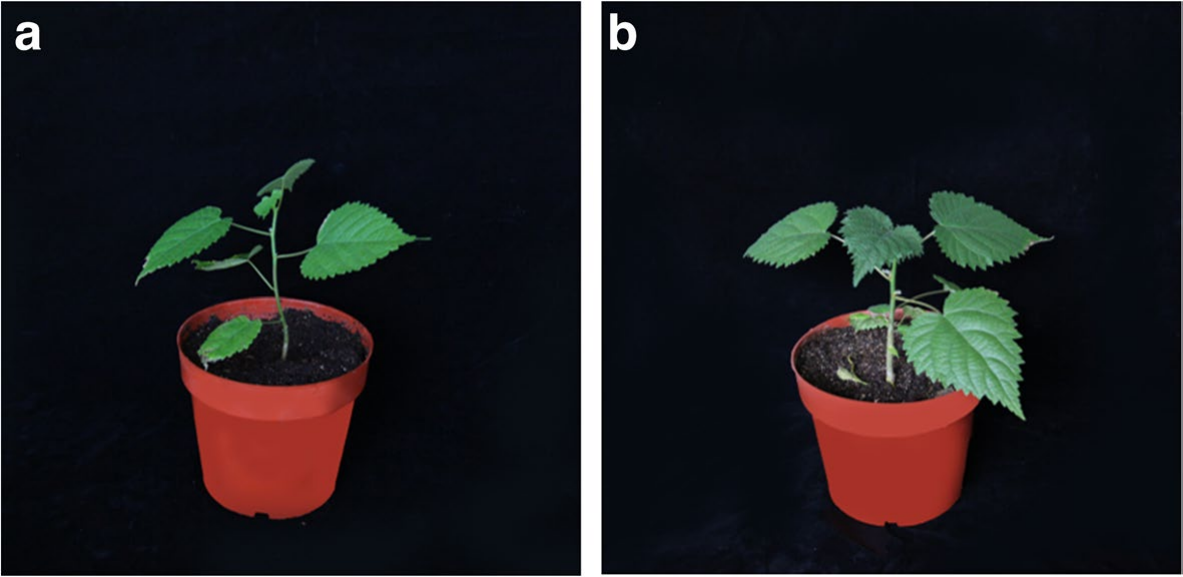
Different ploidy plantlets. **a** diploid, **b** tetraploid

It can be seen from Table 6 that there are phenotypic differences between tetraploid *B. papyrifera* and diploid. The stem of tetraploid *B. papyrifera* is stronger than that of diploid, the length and width of leaves are increased and the node spacing and plant height are shorter (Fig. 3).

**Table 6:** Morphology of *B. papyrifera* with different ploidy

Characteristics	Diploid	Tetraploid	Significance
Ground diameter (mm)	5.55 ± 0.74	7.98 ± 0.24	^*^
Plant height (mm)	133.33 ± 14.59	109.33 ± 5.60	NS
Leaf length (mm)	99.74 ± 8.48	114.12 ± 7.94	NS
Leaf width (mm)	75.41 ± 8.00	100.54 ± 6.83	NS
Length–width ratio of leaf	1.33 ± 0.03	1.14 ± 0.03	^*^
Petiole length (mm)	31.34 ± 5.83	51.92 ± 7.68	NS
Petiole thick (mm)	1.89 ± 0.26	2.89 ± 0.23	^*^
Internodal length (mm)	23.57 ± 1.02	12.86 ± 1.30	^*^

Each value represents the mean ± SE of three replicates

^*^P < 0.05, *NS* represents not statistically significant

#### Stomatal and leaf cell structure

**Fig. 4:**
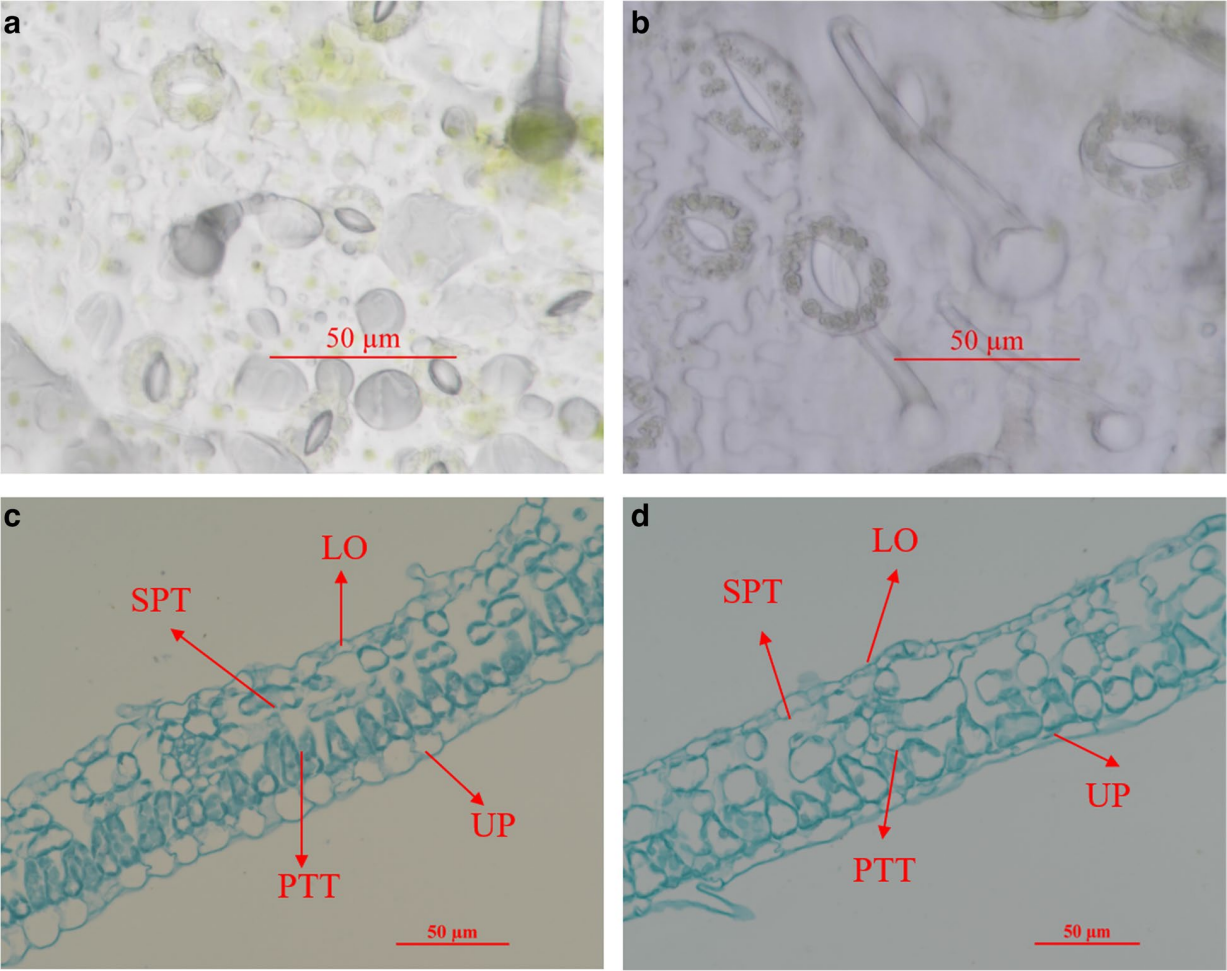
Observation on stomata and leaf cell structure of different ploidy. **a**, **c** diploid; **b**, **d** tetraploid. UP: Upper epidermis thickness; LO: Lower epidermis thickness; SPT: Sponge tissue thickness; PTT: Palisade tissue thickness

The stomata of *B. papyrifera* at different ploidy levels are shown in Fig. 4. According to Table 7, the differences in stomatal length and density between diploid and tetraploid were observed under an optical microscope. The length of stomata in tetraploid was significantly longer than that in diploid, and the density was significantly lower. The observation of leaf anatomical structure showed that the thickness of palisade tissue, the thickness of spongy tissue and the CTR of tetraploid were significantly greater than those of diploid, while the thickness of the upper and lower epidermis of the leaf was lower (Fig. 4).

**Table 7:** Difference analysis of stomata and leaf tissue structure of different ploidy

Characteristics	Diploid	Tetraploid	Significance
Stomata density (mm^−2^)	199.17 ± 13.48	101.79 ± 6.50	^*^^*^
Stomata length (μm)	24.02 ± 1.83	41.27 ± 1.90	^*^^*^
Palisade tissue thickness (μm)	21.03 ± 0.25	25.10 ± 0.24	^*^^**^
Sponge tissue thickness (μm)	22.98 ± 0.28	24.36 ± 0.32	^*^^*^
Ratio of palisade to sponge tissue	0.92 ± 0	1.05 ± 0.01	^*^^**^
Upper epidermis thickness (μm)	11.60 ± 0.14	10.79 ± 0.20	^*^^*^
Lower epidermis thickness (μm)	6.50 ± 0.17	4.81 ± 0.08	^*^^**^
Leaf thickness (μm)	62.73 ± 0.70	62.78 ± 0.48	NS
CTR (%)	33.51 ± 0	40.63 ± 0	^*^^**^

Each value represents the mean ± SE of three replicates

^**^P < 0.01

^***^P < 0.001, *NS* represents not statistically significant

### Photosynthetic features

The observations (Table 8) showed that there were significant differences in the photosynthetic features of *B. papyrifera* with different ploidies. The chlorophyll content (SPAD value) and leaf nitrogen content of tetraploids were higher than diploids. The net photosynthetic rate (*P* < 0.001), stomatal conductance (*P* < 0.001), intercellular CO_2_ concentration (*P* < 0.001) and transpiration rate (*P* < 0.001) of tetraploid were significantly higher than those of diploid, which were 9.61 μmol·m^−2^·s^−1^, 0.13 mol·H_2_Om^−2^·s^−1^, 287.87 μl·L^−1^, 2.78 mmol·m^−2^·s^−1^ respectively.

**Table 8:** Photosynthetic features of different ploidy

Characteristics	Diploid	Tetraploid	Significance
Chlorophyll content (SPAD value)	30.10 ± 0.53	34.40 ± 0.55	^*^^*^
Nitrogen content (mg·g^−1^)	12.17 ± 0.14	13.57 ± 0.18	^*^
Net photosynthetic rate (μmol·m^−2^·s^−1^)	8.17 ± 0.92	9.61 ± 0.83	^***^
Stomatal conductance (mol·H_2_Om^−2^·s^−1^)	0.10 ± 0.02	0.13 ± 0.02	^***^
Intercellular CO_2_ concentration (μl·L^−1^)	268.42 ± 16.10	287.87 ± 9.79	^***^
Transpiration rate (mmol·m^−2^·s^−1^)	2.10 ± 0.35	2.78 ± 0.30	^***^

Each value represents the mean ± SE of three replicates

^*^P < 0.05

^**^P < 0.01

^***^P < 0.001

## Discussion

In this paper, the tetraploid of *B. papyrifera* was successfully induced from leaves, callus and seeds treated with colchicine for the first time, the ploidy was identified by chromosome counting and flow cytometry, and it was found that the induction rate of leaves treated with liquid colchicine was better. It may be that in liquid colchicine, the leaves of *B. papyrifera* are more fully exposed to colchicine, which increases the probability of cell mutation. Previously, polyploid induction of mulberry (trees of the same family as *B. papyrifera*) was reported. Wang et al. [41] treated mulberry regenerated shoots with colchicine by a dip (0.0%, 0.1%, 0.15% and 0.2%) and drip (0.0%, 0.15%, 0.2% and 0.25%), and successfully induced mulberry polyploids.

Moreover, the results showed that the tetraploid induction rate of *B. papyrifera* leaves was higher than that of callus and seeds. This may be the callus and seeds are too sensitive to colchicine, high concentrations of colchicine easily lead to callus and seed inactivation. Relatively, the tolerance of leaves is higher. Of course, different plants have different types of explants. For example, different explants of *Viti*s x *Muscadinia* hybrids were treated with colchicine, and the highest tetraploid induction rate was obtained when the shoot tips were treated with 625 μM liquid colchicine medium for 72 h [42]. Although colchicine can effectively induce polyploidy, it is toxic to plants [43]. The results of this study showed that the regeneration ability of the material decreased significantly with the increase of colchicine concentration and treatment time. Meanwhile, the growth of *B. papyrifera* explants treated with colchicine was relatively slow, which may be due to the physiological interference of colchicine, resulting in a decrease in cell division rate [44]. This is consistent with the research results of polyploid induction of Gerbera (*Gerbera jamesonii* Bolus cv. Sciella) [45], yacon (*Smallanthus sonchifolius*) [46] and Patchouli (*Pogostemon cablin* Benth.) [47]. Colchicine can obtain polyploid by inhibiting the formation of microtubules, but this can also lead to the disintegration of the cytoskeleton in plant cells, resulting in cell death [48]. This paper also found that in addition to tetraploid, colchicine induction also induced chimeras. Because the cell division period of the plant material meristem is not synchronous, the effect of colchicine on the cells of the apical meristem is not consistent, so mixed ploidy plants with diploid cells and tetraploid cells will be formed [49]. Although mixed ploidy may be better than diploid in some characteristics, the ploidy level is unstable, which is not conducive to production and application [50].

The morphological indexes of ground diameter, leaf length, leaf width and petiole length of tetraploid *B. papyrifera* were larger than those of diploid *B. papyrifera*. Studies have shown that after chromosome doubling, gene expression will change due to dose and interaction effects, resulting in morphological and physiological changes. Due to the increase in chromosomes and DNA, cells in polyploid plants enlarge [51]. Therefore, compared with the morphology of diploid plants, polyploid plants have larger vegetative organs, which can be used to screen and distinguish plants with different diploid levels. Compared with diploid plants, many polyploid plants have larger organs, such as larger pollen grains of tetraploid basil [52] and larger leaf mass of tetraploid European pear [53]. However, we also found that the internode length of tetraploid *B.papyrifera* was shorter than that of diploid *B.papyrifera*, and the plant height was shorter. Some scholars have pointed out that this may be because polyploid plants need to consume more materials to grow and maintain DNA, resulting in a decline in some indicators [54]. Our results confirmed that the stomata of polyploid plants were significantly greater than diploid plants, while the stomatal density was the opposite. This is consistent with the research results of *Brachiaria ruziziensis* [55], *Passiflora edulis* Sims [56], Alocasia [57], *Dendranthema nankingense* [58]. The stomatal parameter may be a powerful index of ploidy level change [59, 60]. In this study, the palisade tissue thickness, spongy tissue thickness and leaf tissue structure tightness (CTR%) of tetraploid *B. papyrifera* were significantly higher than those of diploid. The tissue structure of plant leaves is of great significance to the study of plant stress resistance [61, 62]. Previous studies have shown that the increase in palisade tissue thickness and sponge tissue thickness may help plant leaves store more water to improve their drought resistance [63, 64]. Therefore, the drought resistance of tetraploid *B. papyrifera* may be better than that of diploid. It has also been reported that polyploid plants are more drought tolerant than their corresponding diploids, such as *Arabidopsis* [65], and Rangpur lime [66]. Table 8 showed that the chlorophyll content, nitrogen content and net photosynthetic rate of tetraploid were significantly higher than those of diploids. Chlorophyll is a part of the photosynthetic system, which absorbs light energy and ultimately promotes CO_2_ assimilation. And the net photosynthetic rate of plants is an important index to measure plant health and photosynthesis, which affects the accumulation of carbohydrates in plants [67, 68]. These indicators of tetraploid *B. papyrifera* are higher than diploid, indicating that the photosynthetic capacity of tetraploid *B. papyrifera* may be better than diploid. Polyploids not only differ from diploids in morphological indicators, photosynthetic features, and leaf structure, but some reports also indicate that the nutritional content of polyploid plants is also different from that of diploids. For example, tetraploid *Sorghum bicolor* (L.) Mönch had higher protein content [69], and tetraploid *Zea mays* L. × *Zea mays ssp.mexicana* (Schrad.) Kuntze had higher soluble sugar content [70]. Therefore, the nutritional components of tetraploid *B. papyrifera* can be determined in the future to study its feasibility as a forage tree species.

## Conclusions

This study shows that tetraploids can be obtained by treating leaves, calli and seeds of *B. papyrifera* with liquid and solid colchicine. This is the first report of the successful induction of tetraploid *B. papyrifera* and a detailed comparison of diploid and tetraploid *B. papyrifera*. The data indicate that treating leaves with 450 mg·L^−1^ liquid colchicine for 3 d is the optimal condition to obtain polyploid plants. Tetraploids showed differences from their corresponding diploids in morphology, stomata, leaf cell structure and photosynthetic characteristics, including larger leaves, thicker ground diameter, larger stomata, thicker leaves, thicker palisade tissue and sponge tissue, higher net photosynthetic rate and higher stomatal conductance. Overall, the results of this study provide an important technical basis for polyploid breeding of *B. papyrifera*, and make an important contribution to improving varieties and creating new germplasm.

### Supplementary Information

**Additional file 1:** **Table S1.** Variance analysis of leaf explants induced by solid colchicine medium. **Table S2.** Variance analysis of callus explants induced by solid colchicine medium. **Table S3.** Variance analysis of leaf explants induced by liquid colchicine medium. **Table S4.** Variance analysis of callus explants induced by liquid colchicine medium. **Table S5.** Variance analysis of seeds induced by liquid colchicine medium.

## Data Availability

The datasets generated during and/or analysed during the current study are available from the corresponding author on reasonable request.
