# Supplementary material for: Induction of tetraploids in Paper Mulberry (Broussonetia papyrifera (L.) L’Hér. ex Vent.) by colchicine
Source: BMC Plant Biol. 2023 Nov 17;23:574. doi: 10.1186/s12870-023-04487-2 (PMC10655367; doi:10.1186/s12870-023-04487-2)
Supplement: Supplementary file 1 — Additional file 1: Table S1. Variance analysis of leaf explants induced by solid colchicine medium. Table S2. Variance analysis of callus explants induced by solid colchicine medium. Table S3. Variance analysis of leaf explants induced by liquid colchicine medium. Table S4. Variance analysis of callus explants induced by liquid colchicine medium. Table S5. Variance analysis of seeds induced by liquid colchicine medium. [file 12870_2023_4487_MOESM1_ESM.docx]

Table S1 Variance analysis of leaf explants induced by solid colchicine medium

|  | | Sum of squares | Df | Mean squre | F | Pr(>F) |
| --- | --- | --- | --- | --- | --- | --- |
| Colchicine Concentration | Adventitious shoot induction rate | 0.307 | 1 | 0.307 | 29.761 | 0 |
|  | Mixed ploidy induction rate | 0.003 | 1 | 0.003 | 0.579 | 0.45 |
|  | Tetraploid induction rate | 0.003 | 1 | 0.003 | 0.846 | 0.362 |
| Duration | Adventitious shoot induction rate | 0.681 | 1 | 0.681 | 66.101 | 0 |
|  | Mixed ploidy induction rate | 0.005 | 1 | 0.005 | 0.903 | 0.347 |
|  | Tetraploid induction rate | 0.004 | 1 | 0.004 | 1.336 | 0.253 |

Table S2 Variance analysis of callus explants induced by solid colchicine medium

|  | | Sum of squares | Df | Mean squre | F | Pr(>F) |
| --- | --- | --- | --- | --- | --- | --- |
| Colchicine Concentration | Adventitious shoot induction rate | 0.287 | 1 | 0.287 | 20.777 | 0 |
|  | Mixed ploidy induction rate | 0.012 | 1 | 0.012 | 4.139 | 0.047 |
|  | Tetraploid induction rate | 0 | 1 | 0 | - | - |
| Duration | Adventitious shoot induction rate | 0.129 | 1 | 0.129 | 9.356 | 0.004 |
|  | Mixed ploidy induction rate | 0.005 | 1 | 0.005 | 1.808 | 0.185 |
|  | Tetraploid induction rate | 0 | 1 | 0 | - | - |

Table S3 Variance analysis of leaf explants induced by liquid colchicine medium

|  | | Sum of squares | Df | Mean squre | F | Pr(>F) |
| --- | --- | --- | --- | --- | --- | --- |
| Colchicine Concentration | Adventitious shoot induction rate | 0.166 | 1 | 0.166 | 13.486 | 0.001 |
|  | Mixed ploidy induction rate | 0.013 | 1 | 0.013 | 2.137 | 0.15 |
|  | Tetraploid induction rate | 0.005 | 1 | 0.005 | 1.082 | 0.303 |
| Duration | Adventitious shoot induction rate | 0.348 | 1 | 0.348 | 28.243 | 0 |
|  | Mixed ploidy induction rate | 0.004 | 1 | 0.004 | 0.661 | 0.42 |
|  | Tetraploid induction rate | 0.003 | 1 | 0.003 | 0.714 | 0.402 |

Table S4 Variance analysis of callus explants induced by liquid colchicine medium

|  | | Sum of squares | Df | Mean squre | F | Pr(>F) |
| --- | --- | --- | --- | --- | --- | --- |
| Colchicine Concentration | Adventitious shoot induction rate | 0.515 | 1 | 0.515 | 28.382 | 0 |
|  | Mixed ploidy induction rate | 0.025 | 1 | 0.025 | 3.413 | 0.071 |
|  | Tetraploid induction rate | 0 | 1 | 0 | 0.329 | 0.569 |
| Duration | Adventitious shoot induction rate | 0.34 | 1 | 0.34 | 18.718 | 0 |
|  | Mixed ploidy induction rate | 0.016 | 1 | 0.016 | 2.229 | 0.142 |
|  | Tetraploid induction rate | 0.001 | 1 | 0.001 | 0.563 | 0.457 |

Table S5 Variance analysis of seeds induced by liquid colchicine medium

|  | | Sum of squares | Df | Mean squre | F | Pr(>F) |
| --- | --- | --- | --- | --- | --- | --- |
| Colchicine Concentration | Adventitious shoot induction rate | 1.108 | 1 | 1.108 | 125.257 | 0 |
|  | Mixed ploidy induction rate | 0.039 | 1 | 0.039 | 7.651 | 0.009 |
|  | Tetraploid induction rate | 0.01 | 1 | 0.01 | 3.839 | 0.058 |
| Duration | Adventitious shoot induction rate | 0.32 | 1 | 0.32 | 36.156 | 0 |
|  | Mixed ploidy induction rate | 0.033 | 1 | 0.033 | 6.419 | 0.016 |
|  | Tetraploid induction rate | 0.004 | 1 | 0.004 | 1.652 | 0.207 |
